# When sperm age, their RNA code hits a cliff

**DOI:** 10.1038/s44318-026-00743-x

**Published:** 2026-03-17

**Authors:** Noriko Osumi

**Affiliations:** Department of Developmental Neuroscience, Tohoku University Graduate School of Medicine, Sendai, Japan

**Keywords:** Methods & Resources, RNA Biology, Stem Cells & Regenerative Medicine

## Abstract

Recent modification-aware RNA sequencing reveals unexpected shifts in sperm small non-coding RNA profiles during lifespan with functional relevance for gene expression.

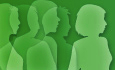

## Teaser

Advanced paternal age is associated with reduced fertility and increased health risks in offspring, but the molecular events that convert “chronological age” into “biological risk” remain incompletely defined. Recent work by Shi et al (2026) uncovers a conserved sharp transition, i.e., an “aging cliff”, in mouse and human sperm small non-coding RNA profiles, suggesting roles of age-dependent remodeling of the sperm epigenetic playload in early embryogenesis and transgenerational outcomes.

For decades, reproductive aging has been framed primarily through the maternal “biological clock.” Yet, delayed fatherhood is now common in many countries, and epidemiology increasingly associates advanced paternal age with reduced fecundity and elevated risks for neurodevelopmental and other complex phenotypes in the next generation (Ashapkin et al, 2023). What has remained less clear is what, exactly, changes in an aging sperm cell that might plausibly connect the father’s life history to embryo development.

The classic explanation has been straightforward: sperm accumulate de novo mutations because spermatogonial stem cells divide continuously over a man’s lifetime. Reviews of germline mutation dynamics and recent high-resolution analyses support this model and refine our understanding of mutational processes in the male germline (Goldmann et al, 2019), which was also validated by more precise DNA sequencing (Neville et al, 2025). However, mutation burden alone rarely accounts for the breadth and variability of reproductive outcomes attributed to older fathers, motivating increased attention to epigenetic and regulatory layers.

Among these layers, sperm small RNAs are particularly compelling because the sperm RNA payload is enriched for small RNA fragments, and these molecules are measurable, dynamic, and well-positioned to influence gene regulation immediately after fertilization (Osumi and Tatehana, 2021; Ashapkin et al, 2023). Earlier work already suggested that paternal aging can shift sperm small-RNA profiles and raised the possibility of links to neurodevelopmental outcomes, although with limited depth and resolution compared with modern sequencing (Miyahara et al, 2023). A major obstacle has been technical: mature sperm are transcriptionally silent, and their RNAs carry modifications that can confound conventional small-RNA sequencing, potentially masking biologically meaningful species.

In this context, the novel study by Shi et al (2026) provides a high-resolution view of sperm RNA aging using PANDORA-seq, designed to reduce modification-induced detection bias and expose otherwise underrepresented sncRNA classes (Shi et al, 2021). Profiling mouse sperm across the lifespan, the authors identify an “aging cliff”, i.e., a sharp transition in the sncRNA landscape, highlighted by coordinated changes in mitochondrial and genomic tRNA-derived small RNAs (tsRNAs) and rRNA-derived small RNAs (rsRNAs). Importantly, they go beyond whole-sperm measurements and examine purified sperm heads, revealing a particularly notable aging signature: an rsRNA length shift, in which longer rsRNAs increase and shorter ones decrease with age. This signature is reproduced in two independent cohorts of aging men, elevating the finding from an age-associated observation in one species to a conserved, cross-species signature.

A key question is causality: do these RNAs merely report aging, or can they plausibly transmit information to the embryo? Shi et al (2026) provide an important functional foothold by showing that tsRNA/rsRNA mixtures resembling aged sperm reprogram gene expression in mouse embryonic stem cells, with enrichment for pathways related to metabolism and neurodegeneration. While such assays do not recreate fertilization, they support the mechanistic proposition that altered sperm sncRNA cargo is sufficient to perturb embryonic-like transcriptional programs. This perspective aligns with the broader conceptual framework that sperm deliver more than a haploid genome and can carry epigenetic information, via chromatin marks and RNAs, that influences offspring development (Osumi and Tatehana, 2021).

These findings arrive amid growing efforts to quantify male reproductive aging using molecular measures rather than chronology. For example, DNA methylation-based sperm epigenetic clocks have been associated with time-to-pregnancy and pregnancy probability, suggesting that sperm “biological age” may capture reproductive risk more effectively than chronological age alone (Pilsner et al, 2022). In parallel, evidence from human embryo studies supports the notion that sperm-borne small RNAs can contribute to early developmental gene regulation, strengthening the plausibility that sperm RNAs are not merely passive remnants but part of the information delivered at fertilization (Isacson et al, 2025).

At the same time, sperm aging is not an RNA-only story. Aging impacts spermatogenesis and epididymal maturation across multiple germ-cell and somatic compartments, contributing to decreased fertility and multi-parameter declines in sperm quality in vivo (Endo et al, 2024). Moreover, multiple epigenetic strata may converge: DNA methylation and chromatin packaging can change with paternal age, and some marks may resist embryonic reprogramming (Ashapkin et al, 2023). Such multilayer remodeling is especially relevant to transgenerational questions, because sperm-borne information could, in principle, influence offspring phenotypes across developmental time.

A timely illustration comes from recent work reporting age-associated DNA methylation alterations at imprint control regions in sperm, proposed to contribute to risk frameworks for autism spectrum disorder in offspring (Casella et al, 2025). These observations remain mechanistically challenging—imprinting is tightly regulated and causal paths to human neurodevelopment require careful triangulation—but they underscore why integrative models are needed: paternal age may reshape the sperm epigenome at multiple layers (small RNAs, methylation, chromatin), and the embryo may interpret these inputs in context-dependent ways (Ashapkin et al, 2023).

Shi et al (2026) therefore provide three advances that move the field forward. First, they define an unexpectedly sharp and measurable transition in sperm sncRNA profiles (the “aging cliff”) using modification-aware sequencing. Second, they identify a conserved human signature at the level of rsRNA length in sperm heads, strengthening its potential as a biomarker candidate. Third, they show that age-mimicking RNA mixtures can reprogram embryonic-like gene expression states, supporting a plausible mechanistic bridge from paternal age to early gene regulation. Next steps include identifying upstream causes of the rsRNA length shift (processing, RNA modifications, mitochondrial signaling, epididymal remodeling), testing links to reproductive endpoints (fertilization, embryo quality, implantation, live birth), and integrating RNA signatures with methylation clocks and physiology to build more accurate, ethically responsible measures of sperm biological age.

If sperm aging includes a phase-like transition in information content rather than smooth drift, it becomes more tractable: cliffs can be mapped, monitored, and perhaps even mitigated. By making sperm sncRNA aging both measurable and functionally interpretable, Shi et al shift the paternal age discussion from an epidemiological association to testable molecular pathways linking the father’s lifespan to early developmental gene regulation in offspring.

## References

[CR1] Ashapkin V, Suvorov A, Pilsner JR, Krawetz SA, Sergeyev O (2023) Age-associated epigenetic changes in mammalian sperm: implications for offspring health and development. Hum Reprod Update 29(1):24–44 36066418 10.1093/humupd/dmac033 PMC9825272

[CR2] Casella E, Depovere J, Delger C, Butynets M, Antczak P, Price T, Jirtle RL, Murphy SK, Hoyo C, Soubry A (2025) Age-specific DNA methylation alterations in sperm at imprint control regions may contribute to the risk of autism spectrum disorder in offspring. Aging 17(12):2950–2988 41474634 10.18632/aging.206348 PMC13147729

[CR3] Endo T, Kobayashi K, Matsumura T, Emori C, Ozawa M, Kawamoto S, Okuzaki D, Shimada K, Miyata H, Shimada K et al (2024) Multiple ageing effects on testicular/epididymal germ cells lead to decreased male fertility in mice. Commun Biol 7:16 38177279 10.1038/s42003-023-05685-2 PMC10766604

[CR4] Goldmann JM, Veltman JA, Gilissen C (2019) De novo mutations reflect development and aging of the human germline. Trends Genet 35(11):828–839 31610893 10.1016/j.tig.2019.08.005

[CR5] Isacson S, Karlsson K, Zalavary S, Asratian A, Kugelberg U, Liffner S, Öst A (2025) Small RNA in sperm–paternal contributions to human embryo development. Nat Commun 16(1):6571 40670377 10.1038/s41467-025-62015-2 PMC12267487

[CR6] Miyahara K, Tatehana M, Kikkawa T, Osumi N (2023) Investigating the impact of paternal aging on murine sperm miRNA profiles and their potential link to autism spectrum disorder. Sci Rep 3(1):20608 10.1038/s41598-023-47878-z PMC10703820 38062235

[CR7] Neville MDC, Lawson ARJ, Sanghvi R, Abascal F, Pham MH, Cagan A, Nicola PA, Bayzetinova T, Baez-Ortega A, Roberts K et al (2025) Sperm sequencing reveals extensive positive selection in the male germline. Nature 647(8089):421–428 41062690 10.1038/s41586-025-09448-3 PMC12611766

[CR8] Osumi N, Tatehana M (2021) Transgenerational epigenetic information through the sperm: sperm cells not just merely supply half of the genome for new life; they also seem to transmit additional information via epigenetic modifications. EMBO Rep 2(8):e53539 10.15252/embr.202153539 PMC8344929 34288347

[CR9] Pilsner JR, Saddiki H, Whitcomb W, Suvorev A, Muck Louis GM, Mumford SL, Schisterman EF, Oluwayiose OA, Balzer LB (2022) Sperm epigenetic clock associates with pregnancy outcomes in the general population. Hum Reprod 37(7):1581–1593 35552703 10.1093/humrep/deac084 PMC9247414

[CR10] Shi J, Zhang X, Cai C, Liu S, Yu J, James ER, Liu L, Emery BR, McMurray Bires MR (2026) Conserved shifts in sperm small non-coding RNA profiles during mouse and human aging. EMBO J 45(4):1362–1380 41559200 10.1038/s44318-025-00687-8 PMC12909834

[CR11] Shi J, Zhang Y, Tan D, Zhang X, Yan M, Zhang Y, Franklin R, Shahbazi M, Mackinlay K, Liu S et al (2021) PANDORA-seq expands the repertoire of regulatory small RNAs by overcoming RNA modifications. Nat Cell Biol 23:424–436 33820973 10.1038/s41556-021-00652-7 PMC8236090

